# Detection of *Theileria equi* and* Babesia caballi* using microscopic and molecular methods in horses in suburb of Urmia, Iran

**Published:** 2014

**Authors:** Farnaz Malekifard, Mousa Tavassoli, Mohammad Yakhchali, Reza Darvishzadeh

**Affiliations:** 1 *Department of Pathobiology, Faculty of Veterinary Medicine, Urmia University, Urmia, Iran; *; 2 *Department of Agronomy and Plant Breeding, Faculty of Agriculture, Urmia University, Urmia, Iran.*

**Keywords:** *Babesia caballi*, Horse, Iran, Multiplex PCR, *Theileria equi*

## Abstract

Equine piroplasmosis is a severe disease of horses caused by the intra-erythrocyte protozoan, *Theileria equi* and *Babesia caballi*. The aim of this study was to identify equine piroplasmosis based on molecular and morphometrical features in horses in suburb of Urmia, West Azerbaijan province, Iran. From April to September 2011, a total number of 240 blood samples were collected randomly from horses of 25 villages. The specimens were transferred to the laboratory and the blood smears stained with Geimsa, and the morphological and biometrical data of parasite in any infected erythrocyte were considered. Extracted DNA from each blood sample was used in multiplex PCR in order to confirm the presence of *B. caballi* and *T. equi*. Microscopic observation on 240 blood smears determined that 15 (6.25%) and 5 (2.80%) samples were infected by *T. equi *and *B. caballi, *respectively. The mixed infections occurred in 2 (0.83%) samples. The results of the PCR assays showed 26 (10.83%), 14 (5.83%) and 4 (1.66%) were distinguished as *T. equi,* *B. caballi *and mixed infection, respectively. Differences in infection rates were statistically nonsignificant between male and female horses and among different age groups. Our findings indicated that *T. equi* and *B. caballi* were prevalent in horse population.

## Introduction

Equine piroplasmosis is a tick-borne disease caused by intra erythrocyte protozoa, *Babesia equi* (recently re-classified as *Theileria equi*)^1^ and *Babesia caballi*.^2^ It is characterized by fever, anemia, icterus, hepatomegaly, edema, intra-vascular hemolysis, hemoglobinuria and even death.^3^^,^^4^ These parasites affect horse industry worldwide, causing economic loss and impacting the international movement of horses.^5^ This disease is distributed in Asia, Europe, Africa and South America. Prevalence of disease is related to distribution of vector ticks.^6^^,^^7^ Tick species of the genera *Boophilus,* *Dermacentor*, *Hyalomma* and *Rhipicephalus *are the biological vectors of equine piroplasmosis.^8^ The clinical picture of piroplasmosis is variable and often nonspecific.^9^ It is not possible to distinguish between *T. equi *and *B. caballi *infections based on clinical signs alone. Several studies have documented mixed infections of *T. equi *and *B. caballi*.^10^^,^^11^ Recovered horses from acute phase of infection serve as reservoirs for both *Babesia* species.^6^

*Theileria equi* is a small piroplasm whereas *B. caballi* is a larger form. The shape of *T. equi* parasite in the infected erythrocyte varies from spherical, ovoid or Maltese cross shape. The organism may be found either singly, in pairs, or in tetrads*. Babesia* *caballi* organisms are pyriform round or oval in shape and commonly seen singly or in pairs.^3^^,^^12^

Equine *Babesia* species detection was performed traditionally using Giemsa staining of thin blood smears and their morphology in infected erythrocytes. This method may have been accompanied with some technical problems.^13^ Recently, several studies have been conducted to describe biometrical and genetical characterization of babesiosis in Iran.^13^^,^^14^

Several sero-epidemiological studies concerning equine babesiosis have been conducted among horses of many parts of the world. The lack of the specificity due to cross reactivity with other species of *Babesia *has been observed in serological investigations.^15^ Molecular techniques have been considered as perfect methods for detection of many species of *Babesia* and *Theileria. *Reportedly, PCR assays have higher sensitivity and specificity compared with serological assays.^16^^-^^18^

The presence of equine piroplasmosis has previously been reported from different parts of Iran. These studies have been performed by microscopic examination and serological methods.^19^^-^^23^ The aim of this study was to identify equine piroplasmosis based on molecular techniques and morphometrical indices in horses in suburb of Urmia, West Azerbaijan province, Iran. 

## Materials and Methods

**Study area. **The study was conducted during the tick activity seasons (spring and summer) in 25 villages of Urmia suburb, capital of West Azerbaijan province. Urmia is semi-humid, with mean precipitation of 350 mm, maximum monthly temperature of 28.3 ˚C in August and minimum monthly temperature of –5 ˚C in January. This area has borders with Turkey and Iraq and some residents of the area usually travel and carry goods by working horses across the borders due to the arduous mountain routes.^24^

**Sampling and morphometric procedures. **From April to September 2011, a total number of 240 blood samples were collected randomly from horses in the mountainous, mountainside, and plain areas of Urmia suburb. Blood samples were aseptically obtained from the jugular vein of each horse. The age and sex were recorded for each animal. The blood samples were collected in the presence of the EDTA anticoagulant and used immediately for blood smears stained with Giemsa. The samples were transferred to the laboratory of Parasitology, Faculty of Veterinary Medicine, Urmia University, Urmia, for further analysis. 

**Giemsa staining. **The Giemsa stained blood smears were examined to determine the presence of hemoprotozoal parasites. The morphological and biometrical parameters including the shape, site location and size of parasite in any infected erythrocyte have been considered.^14^ In microscopic examination, *B. caballi *was identified as large paired pyriform parasites, while the small *T. equi *parasites were identified as paired pyriform, rounded and tetrad or Maltese cross arrangement of merozoites.^12^^,^^25^

**DNA extraction. **Genomic DNA was extracted according to Alhassan *et al*. with some modifications.^11^ Briefly, 50 μL of each horse blood samples were washed three times with cold phosphate buffered saline by centrifuging at 1000 *g* for 5 min at 4 ˚C and re-suspending in 100 µL of DNA extraction buffer (0.1 mM Tris-HCl [pH 8.0], 0.1% sodium dodecyl sulfate, 100 mM NaCl, 10 mM EDTA, and 100 μg mL^-1^ proteinase K) and incubating at 55 ˚C for 2 hr. The parasitic DNA was extracted with phenol-chloroform and precipitated with ethanol. The purified DNA pellets were dissolved in 20 μL of double-distilled water for subsequent PCR reactions.^11^

**Multiplex PCR. **In order to specify the morphological findings and simultaneous differentiation of *B. caballi *and* T. equi*, multiplex PCR based on the 18S ribosomal RNA genes was performed. A set of primers, Bec-UF2 5´-TCGAAGACGATCAGATACCGTCG-3´, Cab-R 5´-CTCGTT CATGATTTAGAATTGCT-3´and Equi-R 5´-TGCCTTAAAC TTCCTTGCGAT-3´, were used to amplify DNA fragments of 540 and 392 bp from *B. caballi *and *T*. *equi, *respectively. The primer’s specificity and sensitivity and also the PCR condition had been described previously by Alhassan *et al*.^11^

The PCR reaction was performed in 50 μL of a mixture (10 mM Tris–HCl [pH 8.3], 50 mM KCl, and 1.5 mM MgCl_2_) containing 3 μL of the template DNA, 2.5 pmol of each of the primers, 0.2 mM dNTP mixture and 2.5 U of *Taq *DNA polymerase (Fermentas, Schwerte, Germany). Cycling condition was 96 ˚C for 10 min, followed by 40 cycles at 96 ˚C for 1 min, 60.5 ˚C for 1 min, and 72 ˚C for 1 min with a final extension step of 72 ˚C for 10 min. The PCR products were analyzed by 1.5% agarose gel electrophoresis, followed by ethidium bromide staining and photography.^11^ Positive controls were consisted of DNA from blood samples known to be infected by *B. caballi *and* T. equi* through microscopic examination of blood smears. Distilled water was used as negative control in PCR amplification.

**Statistical analysis. **Data were analyzed using SPSS (Version 17; SPSS Inc., Chicago, USA). A value of *p* < 0.05 was considered as statistically significant.

## Results

Out of 240 examined horses, 129 were females and 111 males. The number of infected horses based on age and sex was summarized in Table 1. Prevalence of *B. caballi* and *T. equi* in all age groups and between male and female horse were not statistically significant (*p > *0*.*05). 

**Table 1:** The frequency (positive/examined) of *Babesia s*pp. infection in horses based on age and sex

**Methods**	**Age (%)**		**Sex (%)**	
	**< 3**	**≥ 3**	**Female**	**Male**
**Microscopy**	9/106 (8.49)	13/134 (9.70)	12/129 (9.30)	10/111 (9.00)
**PCR**	19/106 (17.92)	25/134 (18.65)	23/129 (17.82)	21/111 (18.91)

**Morphological and morphometric findings. **Microscopic observation on 240 blood smears determined that 15 (6.25%) and 5 (2.80%) samples were infected by *T. equi *and *B. caballi*, respectively. The mixed infections were occurred in 2 samples (0.83%). The parasites shapes were distinguished based on single round, double round, single pyriform and double pyriform with obtuse or acute angle. The size of *T. equi* and *B. caballi* typical paired pyriforms and round forms are summarized in Table 2.

**Table 2:** The morphological features of* T. equi* and *B. caballi*. The data are presented as mean ± standard deviation

**Parasite**	**Morphological feature**		**Size (μm)**
***T*** ***. *** ***equi***	Double pyriform - acute angle	1.14 ± 0.15 × 1.40 ± 0.11	
	Double pyriform - obtuse angle	1.60 ± 0.07 × 1.88 ± 0.13	
	Round	1.5 ± 0.19	
***B*** ***. *** ***caballi***	Double pyriform - acute angle	2.60 ± 0.08 × 2.88 ± 0.11	
	Double pyriform - obtuse angle	3.53 ± 0.14 × 3.91 ± 0.07	
	Round	2.53 ± 0.28	

**Fig. 1:**
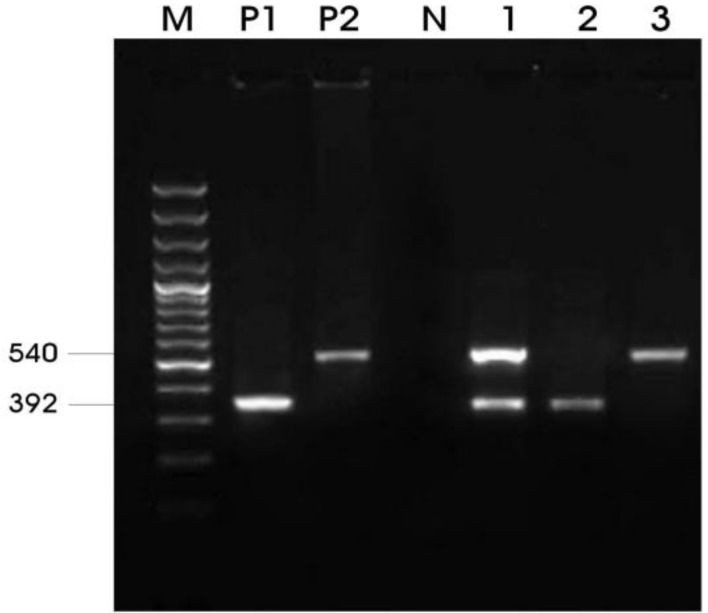
PCR detection of *B. caballi* and *T. equi* with a set of primer combinations (Bec-UF2, Cab-R, and Equi-R). M = 100 bp DNA marker; P1= Positive control for *T. equi*; P2 = Positive control for* B. caballi*; N= Negative control; Lane 1 = Mixed of *T. equi *and* B. caballi*; Lane 2 =* T. equi*; Lane 3 = *B. caballi*

**Molecular findings. **The results of the PCR assays showed, 26 (10.83%) and 14 (5.83%) were infected with *T. equi* and *B. caballi*, respectively. The PCR product of *T. equi* and *B. caballi* were 392 bp and 540 bp, respectively. A mixed infection of *B. caballi* and *T. equi* was found just in 4 horses (1.66%), (Fig.1). 

## Discussion

Visual detection of piroplasms by microscopic examination as the simplest and most accessible diagnostic test, confirms the clinical diagnosis of the babesiosis.^26^ Considering the possible falsely diagnosed cases of babesiosis, the combination of microscopy and PCR based diagnostics is recommended.^27^

Equine piroplasms can be recognized based on biometrical and morphometric data. Soulsby described* T. equi *as small *Babesia *being 2 μm in length (< 2.5 μm), pyriform or comparatively rare round or amoeboid and *B.* *caballi *as a large *Babesia *measuring 2.5 to 4 μm (> 2.5 μm) with acute angle in pyriform. The morphological characteristics observed in *T.equi* and *B.caballi* in current study was in agreement with the findings of Soulsby.^28^

Previous research focused on diagnosis using microscopic examinations of Giemsa-stained blood smears has been reported infection rates of *T. equi* varying 3.50 to 7.00% in Iran.^19^^,^^21^ In the present study, 6.25, 2.80 and 0.83% samples were infected by *T. equi*,* B. caballi* and mixed infections, respectively. Microscopic examination of Geimsa stained blood smears is the common method for diagnosis of these piroplasms in Iran. The low sensitivity of this method does not permit its use in epidemiological investigations.^14^ The results of this study confirmed findings of Bashiruddin *et al*. in that PCR is more sensitive in diagnosing piroplasmosis than microscopy.^29^

In our study no differences were observed between the *T. equi* and *B. caballi* prevalence in all age and sex groups of the horse examined. It may be due to high number of ticks in this area and continuous exposure of young and old horses to infected ticks.^30^

The results of molecular and microscopic examinations confirmed the simultaneous infection of horses in the study region with both equine *Babesia *species, which was consistent with findings of Seifi *et al*. and Abedi *et al*. They reported mixed infection of *T. equi* and *B. caballi* in horses of Turkmen region in Iran.^19^^,^^22^

*Theileria equi* is more common and pathogenic than *B. caballi* in endemic countries.^31^^-^^33^ The results of the present study demonstrated that *T. equi *was more prevalent than *B. caballi*. Our findings were in agreement with the previous study in Iran.^19^ A possible reason for the low prevalence of *B. caballi* could be associated with the earlier removal of the parasite after a short term of infection.^34^

Several investigations on the prevalence of equine piroplasmosis in Turkey, which shares a border with the study area, have been published.^35^^-^^38^ These reports demonstrated that both *T. equi* and *B. caballi *infections in horses have been widespread in Turkey, with the prevalence rate of 7.00% and 3.00%, respectively.^38^

Because of certain geographical specifications of studied area, the sampled horses had close communication with horses of neighboring countries and probably, they had been contentiously exposed to ticks and protozoa. This situation emphasizes the importance of border control and quarantine.^35^ Probably, an increase in the number of imported horses from neighboring countries and the distribution of vector ticks are factors which increase the occurrence of infection in this region. 

Based on our results, it is concluded that *T. equi* and *B. caballi* were prevalent among horses in West Azerbaijan province, Iran. Moreover, this report suggests the possibility of an endemic nature of equine piroplasmosis in this area. These data are essential to establish adequate control measures in this area. The tick vectors for equine piroplasmosis in studied region are still unknown to date, therefore, there is a need to investigate the potential tick vectors involved in the transmission of both *T. equi *and *B. caballi *in horses in this region.

## References

[1] Mehlhorn H, Schein E (1998). Redescription of Babesia equi Laveran, 1901 as Theileria equi Mehlhorn, Schein 1998. Parasitol Res.

[2] Alhassan A, Govind Y, Thanh NT (2007). Comparative evaluation of the sensitivity of LAMP, PCR and in vitro culture methods for the diagnosis of equine piroplasmosis. Parasitol Res.

[3] Schein E, Ristic M (1988). Equine babesiosis. Babesiosis of Domestic Animals and Man.

[4] Uilenberg G (2006). Babesia-a historical overview. Vet Parasitol.

[5] Friedhoff KT, Tenter AM, Muller I (1990). Hemoparasites of equines: Impact on international trade of horses. Rev Sci Tech.

[6] De Waal DT (1992). Equine piroplasmosis: A review. Br Vet J.

[7] Friedhoff KT, Soule C (1996). An account on equine babesiosis. Rev Sci Tech.

[8] Battsetseg B, Xuan X, Ikadai H (2001). Detection of Babesia caballi and Babesia equi in Dermacentor nuttalli adult ticks. Int J Parasitol.

[9] Zobba R, Ardu M, Niccolini S (2008). Clinical and laboratory findings in equine piroplasmosis. J Equine Vet Sci.

[10] Xu Y, Zhang S, Huang X (2003). Seroepidemiologic studies on Babesia equi and Babesia caballi infections in horses in Jilin province of China. J Vet Med Sci.

[11] Alhassan A, Pumidonming W, Okamura M (2005). Development of a single-round and multiplex PCR method for the simultaneous detection of Babesia caballi and Babesia equi in horse blood. Vet Parasitol.

[12] Kuttler KL, Ristic M (1988). Chemotherapy of babesiosis. Babesiosis of domestic animals and man.

[13] Shayan P, Hooshmand E, Nabian S (2008). Biometrical and genetical characterization of large Babesia ovis in Iran. Parasitol Res.

[14] Sadeghi Dehkordi Z, Zakeri S, Nabian S (2010). Molecular and biomorphometrical identification of ovine babesiosis in Iran. Iranian J Parasitol.

[15] Persing DH, Herwaldt B L, Glaser C (1995). Infection with a Babesia like organism in northern California. N Engl J Med.

[16] Geysen D, Delespaux V, Geerts S (2003). PCR-RFLP using Ssu-rDNA amplification as an easy method for species-specific diagnosis of Trypanosoma species in cattle. Vet Parasitol.

[17] Buling A, Criado-Fornelio A, Asenzo G (2007). A quantitative PCR assay for detection and quantification of B bovis and B bigemina. Vet Parasitol.

[18] Jefferies R, Ryan UM, Irwin PJ (2007). PCR-RFLP for the detection and differentiation of the canine piroplasm species and its use with filter paper-based technologies. Vet Parasitol.

[19] Abedi V, Razmi GH, Seifi H (2012). Survey of piro-plasmosis in horses of Turkoman breed horses by serological methods.

[20] Aslani M (1996). One case study of Babesia caballi in horse.

[21] Mohammadzade H Survey of infection of equids with microfillers in Urmia region. DVM thesis.

[22] Seifi HA, Mohri M, Sardari K (2000). A mixed infection of Babesia equi and Babesia caballi in a racing colt: A report from Iran. J Equine Vet Sci.

[23] Sakha M (2007). Successful treatment of babesiosis in a horse. J Vet Res.

[24] Tavassoli M, Dalir-Naghadeh B, Esmaeili-Sani S (2010). Prevalence of gastrointestinal parasites in working horses. Pol J Vet Sci.

[25] Levine ND (1971). Taxonomy of the piroplasms. Trans Am Micros Soc.

[26] Irwin PJ (2010). Canine babesiosis. Vet Clin North Am Small Anim Pract.

[27] Kubelová M, Sedlák K, Panev A (2013). Conflicting results of serological, PCR and microscopic methods clarify the various risk levels of canine babesiosis in Slovakia: A complex approach to Babesia canis diagnostics. Vet Parasitol.

[28] Soulsby EJL (1982). Helmints, arthropods and protozoa of domesticated animals.

[29] Bashiruddin JB, Cammà C, Rebêlo E (1999). Molecular detection of Babesia equi and Babesia caballi in horse blood by PCR amplification of part of the 16S rRNA gene. Vet Parasitol.

[30] Razmi GR, Naghibi A, Aslani MR (2002). An epidemio-logical study on ovine babesiosis in the Mashhad suburb area, province of Khorasan, Iran. Vet Parasitol.

[31] De waal DT (1995). Distribution, transmission and sero-diagnosis of Babesia equi and Babesia caballi in South Africa. PhD Thesis.

[32] Zweygarth E, Lopez-Rebollar LM, Nurton J (2002). Culture, isolation and propagation of Babesia caballi from naturally infected horses. Parasitol Res.

[33] De waal DT (2000). Global importance of piroplasmosis. J Protozool Res.

[34] Salim BO, Hassan SM, Bakheit MA (2008). Diagnosis of Babesia caballi and Theileria equi infections in horses in Sudan using ELISA and PCR. Parasitol Res.

[35] Akkan HA, Karaca M, Tutuncu M (2003). Serologic and microscopic studies on babesiosis in horses in the eastern border of Turkey. J Equine Vet Sci.

[36] Balkaya I, Erdogmus SZ (2006). Investigation of prevalence of Babesia equi (Laveran, 1901) and Babesia caballi (Nuttall, 1910) in horses by serological methods in Elazig and Malatya province. Firat Uni J Health.

[37] Piskin C, Deniz A, Utuk AE (2008). Seroprevalance of dourine and equine piroplasmosis in horses between the years 2002-2007 in Turkey.

[38] Güçlü HZ, Karaer KZ (2007). Detection of Babesia caballi (Nuttall, 1910) and Theileria equi (syn Babesia equi, Laveran, 1901) by polymerase chain reaction (PCR) in show and sport horses in the region of Ankara. Turkiye Parazitol Derg.

